# 

**Published:** 1873-06

**Authors:** 


### Contents of June Number.

### ORIGINAL DEPARTMENT.

ART. I.—Etiology of Bright's Disease. By F. C. Curtis, M. D., Albany, N. Y. ..... 401
ART. II.—Hospital Hygiene. By J. De Zouche, M. D., Albany, N. Y... 406
ART. III.—Medical Society of the County of Albany. Semi-Monthly Meeting, May 14, 1873. Reported by F. C. Curtis, M. D. ..... 413
ART. IV.—Abstract of the Proceedings of the Buffalo Medical Association, April 1st, 1873. ..... 420

### MISCELLANEOUS.

Pancreatic Juice as a Therapeutical Agent ..... 425
Injection of Uric Acid ..... 426
Simon on Artificial Dilatation of the Anus ..... 427
Fluid Extract of Male Fern in Tape Worm ..... 428
Suppression of Perspiration ..... 428
Contribution to the Study of Puerperal Septicæmia ..... 429
Enucleation of Uterine Febroids ..... 430
Some attempts at Human Anaplasia by means of Mucous Grafts from the Cheeks and Tongues of Rabbits and Oxen ..... 431

### EDITORIAL DEPARTMENT.

Popular Lectures upon Medical Subjects ..... 433
Stevens Triennial Prize ..... 434
Toner Lectures ..... 435
Items in Brief ..... 436

BOOKS REVIEWED:
Civil Malpractice. By M. A. McClelland, M. D. ..... 438
Notices of New Publications ..... 439
Books and Pamphlets Received ..... 440

THE BUFFALO

